# The sympathetic baroreflex across the lifespan: Is loss of hysteresis a stop on the road to dysfunction?

**DOI:** 10.1113/EP094341

**Published:** 2026-09-30

**Authors:** Maria C. R. G. Trindade, Qudus A. Ojikutu, Igor A. Fernandes

**Affiliations:** ^1^ Human Neurovascular Control Laboratory, Department of Health and Kinesiology Purdue University West Lafayette Indiana USA; ^2^ Division of Respiratory Diseases, Department of Medicine, Paulista School of Medicine Federal University of São Paulo São Paulo Brazil

**Keywords:** baroreflex and hysteresis, muscle sympathetic nerve activity

Blood pressure rises with advancing age, as does sympathetic vasoconstrictor activity. Yet the two do not march in lockstep. Individuals with comparable blood pressures can exhibit remarkably different levels of muscle sympathetic nerve activity (MSNA), illustrating that the age‐related rise in sympathetic outflow is only one part of a much more complex reorganization of blood pressure control. At the centre of this regulation is the arterial sympathetic baroreflex, our principal beat‐to‐beat defence against blood pressure fluctuations. Importantly, the reflex can be viewed as a series of interconnected steps: a mechanical component, whereby changes in blood pressure deform the barosensory vessels, followed by a neural component, whereby this deformation is translated through afferent signalling, central integration and efferent sympathetic activity into changes in MSNA. The interaction between these components ultimately determines the integrated sympathetic baroreflex response. But does this system simply become progressively weaker as we age? Evidence suggests a more intriguing possibility. Ageing appears to reshape different components of the reflex at different rates. Using the modified Oxford technique, Studinger et al. (2009) showed that older adults have reduced mechanical transduction of pressure into carotid deformation, yet greater neural responsiveness may partially counteract this mechanical deficit. Adding another layer to this complexity, the sympathetic baroreflex does not respond identically when blood pressure falls and rises. In healthy young adults, falling pressure elicits greater sympathetic responsiveness than rising pressure – a phenomenon termed baroreflex hysteresis that may favour rapid sympathoexcitation when blood pressure is threatened. With older age, however, this directional asymmetry becomes attenuated (Studinger et al., 2009). These observations challenge the simple view of baroreflex ageing as a gradual loss of sensitivity. Instead, the journey from young adulthood to old age may involve a succession of adaptations, with one component changing while another compensates to preserve the integrated response. But where along this road do the first changes appear? And, perhaps more importantly, does adaptation inevitably become dysfunction? In this context, the study by Hughes et al. (2026) in this issue of *Experimental Physiology* brings these questions to an especially revealing – and comparatively understudied – stop along the lifespan: middle age.

Hughes et al. (2026) provide an important new piece of this puzzle by examining the mechanical and neural components of sympathetic baroreflex hysteresis during spontaneous, beat‐to‐beat fluctuations in blood pressure. This distinction is relevant because previous work using the modified Oxford technique interrogated the reflex across relatively large, pharmacologically induced changes in blood pressure. In contrast, the spontaneous approach asks how the baroreflex behaves within the narrower pressure range in which it operates from beat to beat. What emerged is intriguing. Middle‐aged men maintained mechanical baroreflex gain and overall integrated burst‐probability gain, yet the pronounced hysteresis of sympathetic burst recruitment observed in young men was no longer evident. The reflex had changed, but it had not obviously failed. Moreover, the change appeared to reside predominantly within the neural rather than the mechanical component: young men responded more strongly to falling than rising pressures, whereas this directional distinction was lost by middle age. Yet the story becomes even more interesting when sympathetic burst strength is considered. Burst‐area hysteresis remained evident in middle‐aged men, and neural burst‐area gain was greater, rather than smaller, than in young men. Given the higher resting MSNA in middle age, one possibility is that further recruitment of sympathetic bursts becomes increasingly constrained, such that modulation of burst strength provides an alternative means of maintaining sympathetic support of blood pressure. Such redistribution of sympathetic coding could become particularly important later in life, when a given sympathetic burst's ability to raise blood pressure is itself attenuated (Vianna et al., 2012). Thus, Hughes et al. may have captured an early stage of baroreflex ageing in which directional neural control is being reorganized while integrated sensitivity remains preserved. Importantly, however, this ‘middle‐age stop’ has so far been mapped only in men. Whether women follow the same trajectory – and whether the timing or nature of this transition is influenced by the profound changes in hormonal milieu that accompany the menopausal transition – remains unknown. The middle‐age stop on the road to baroreflex dysfunction may therefore be characterized less by loss of the reflex itself than by a change in the strategy used to maintain it, but whether men and women arrive at this stop by the same route remains to be established.

This interpretation exposes an important paradox: if integrated baroreflex sensitivity remains intact, does loss of hysteresis actually matter? The answer is not yet clear, and this uncertainty may be the study's most important contribution. A preserved integrated slope does not necessarily mean that the reflex operates normally, just as an altered component does not necessarily indicate dysfunction. More fundamentally, the ‘neural component’ measured by Hughes et al. encompasses a large physiological territory – from baroreceptor afferent signalling and central integration to the recruitment and strength of postganglionic sympathetic discharge. Where, then, is hysteresis being lost? Identifying where along this pathway ageing first alters directional processing is an important next step. Simultaneous MSNA and functional magnetic resonance imaging already allows spontaneous sympathetic bursts to be linked to activity within the nucleus tractus solitarius, caudal ventrolateral medulla and rostral ventrolateral medulla in conscious humans (Macefield & Henderson, 2025), providing an exciting opportunity to determine whether loss of peripheral baroreflex hysteresis is accompanied by altered directional processing within the central baroreflex network. Neurochemical imaging may eventually add another dimension. Proof‐of‐concept experiments in animals have linked dorsomedial medullary neurotransmitter profiles measured using proton magnetic resonance spectroscopy to spontaneous baroreflex function, raising the possibility that functional and neurochemical imaging could ultimately help distinguish altered afferent signalling from changes in central neurotransmission (Garcia‐Espinosa et al., 2010). Yet the road should also be followed in the opposite direction. MSNA is not the final output of the baroreflex: sympathetic activity must be transduced into vasoconstriction and, ultimately, a change in blood pressure. Future studies that follow the entire reflex – from pressure and arterial deformation, through central neural processing and sympathetic discharge, to vascular transduction – in both women and men across young, middle‐aged and older adulthood will be necessary to establish whether loss of hysteresis represents early dysfunction, successful compensation or perhaps both at different stages of life. The road to baroreflex dysfunction may begin long before conventional measures tell us that the reflex is impaired. The critical stop may be the one at which the system has already changed, but compensation still works.

## AUTHOR CONTRIBUTIONS

Maria C. R. G. Trindade, Qudus A. Ojikutu and Igor A. Fernandes conceived the work, drafted the manuscript, revised it critically for important intellectual content and approved the final version. All authors have read and approved the final version of this manuscript and agree to be accountable for all aspects of the work in ensuring that questions related to the accuracy or integrity of any part of the work are appropriately investigated and resolved. All persons designated as authors qualify for authorship, and all those who qualify for authorship are listed.

## CONFLICT OF INTEREST

None declared.

## GENERATIVE AI STATEMENT

Grammarly Pro (version 1.187.0.0, WebUI 2.17.7; Grammarly, Inc.) was used solely to assist with English‐language editing, grammar, and stylistic refinement. The tool identified grammatical errors and suggested improvements to sentence clarity and style. The authors reviewed and approved all suggested changes. Generative AI was not used to generate scientific content or findings, analyse or interpret data, create references, or formulate the scientific conclusions.
